# Effect of yogic education system and modern education system on sustained attention

**DOI:** 10.4103/0973-6131.53840

**Published:** 2009

**Authors:** R Rangan, H R Nagendra, Ramachandra Bhatt

**Affiliations:** Department of Yoga Research, Swami Vivekananda Yoga Anusandhana Samsthana, Bangalore - 560 019, India

**Keywords:** Gurukula education system, sustained attention, vedic chanting; yoga

## Abstract

**Background/Aim:**

Sustained attention is a vital function mediated by the right frontoparietal cortex. The Six Letter Cancellation Task (SLCT) measures sustained attention. Development of sustained attention in a yoga-based education system compared to a modern one is the theme of the present study. Aim: To compare the effectiveness of the Modern Education System (MES) and the Gurukula Education System (GES) in developing sustained attention.

**Materials and Methods:**

Forty nine boys (11-13 years) were selected from two residential schools, one MES and the other GES, providing similar ambiance and daily routines. The boys were matched for age and socioeconomic status. The GES educational program is based around integrated yoga modules while the MES provides a conventional modern education program. Sustained attention was assessed using the SLCT at the start and end of an academic year.

**Results:**

Within groups, the pre-post test differences were significant for both groups. However, the between groups result showed improvement in the GES group compared to the MES group at a *P* < 0.001 significance level.

**Conclusions:**

The study suggests that both MES and GES improve sustained attention in school boys, but GES is more effective.

## INTRODUCTION

This paper is a study of improvements in sustained attention over a period of one academic year in two groups of students, one studying under the traditional Gurukula Education System (GES), and the other under the Modern Education System (MES). Attention is an essential element of cognition and has been characterized in two ways, that is, either as a resource or capacity or as a skill of resource deployment. Sustained attention is the capacity to attend to a task in hand for a required period of time. It is closely associated with task difficulty or complexity. Sustaining attention is easier for simple tasks than complex tasks. It is closely associated with the mental effort required by the task in hand.[1] The capacities to study and listen to a lecture for an extended length of time are examples of sustained attention.

Various brain areas mediate attention, different ones being responsible for different types of attention. The right frontoparietal area mediates sustained attention. Damage to the right prefrontal cortex is associated with poor sustained attention.[2] Imaging studies have shown that vigilance tasks requiring sustained attention activate a network of neurons in the right frontal and parietal cortices.[3]

Many papers have been published analyzing the effect of different aspects of yoga including physical postures and meditation on sustained attention. Special physical postures (*asanas*), voluntary regulation of breathing (*pranayama*), maintaining silence, and visual focusing exercises (*tratakas*) improve attention span in school children.[4]. The Self-Regulation Method (SRM) derived from autogenic training and Zen meditation, which elicits a state of ‘relaxed alertness’ also increases attention span.[5] Meditation increases attention span whereas eyes-closed rest without meditation does not[6]

In a previous study at sVYASA, Sarang *et al*, assessed performance on the Six Letter Cancellation Task (SLCT) – a task requiring visual selectivity and repetitive motor response – in forty male subjects immediately before and after two yoga-based relaxation techniques of equal duration, that is, Cyclic Meditation (CM) and Supine Rest (SR).[7] CM consists of alternating cycles of yoga postures and SR. Both practices significantly improved net scores (*P* < 0.001), CM producing more change (26%) than SR (14%). These results suggest that CM brings about a greater improvement in task performance. The study indicates that yoga improves sustained attention.

The GES is a system of learning based on the Vedas, which includes many yogic practices. It is time-tested and has been preserved for several millennia in an unbroken tradition. In Indian tradition, it is believed that the GES brings great benefits to society, including improvements in cognitive and higher mental abilities.[1] Although the GES stands at the heart of the tradition, scientific examination and understanding of its advantages has not been adequately documented. Hence, the present work was designed to undertake a comparative study of the effect of the GES and MES on sustained attention in boys over a single academic year.

## MATERIALS AND METHODS

### Subjects

Two residential schools (one GES (Prabodhini Gurukula, Ajeya Vishvastha Mandali, Hariharapura, Koppa taluk, Chikmangalore district, Karnataka.) and the other MES (Indian Matriculation Higher Secondary School, Gopinathanpatti, X-road, Palaya Patti, Pudur Post, Pappi Retti Patti Taluk, Dharmapuri district, Tamilnadu.) providing similar ambiance and daily routines were selected for the study. Both schools were residential, and had similar natural surroundings with an atmosphere congenial for learning. Out of 110 students studying in the yoga-based GES and 500 students studying in MES, 49 healthy boys (11–13 years) from each school were matched for age, family atmosphere, and socioeconomic background. Their health status was assessed based on their personal history and a general clinical examination. Any on medication known to affect planning or cognitive abilities were excluded from the study. The students in the GES school were all freshers and had received modern education prior to joining the GES school. An independent samples ‘t’ test on the baseline data as described in Table 1, showed no significant differences (*P* > 0.05) between the two groups for any of the demographic parameters.

**Table 1:** Demographic data of boys studying in GES and MES schools

Groups	Number of students *n*	S (Mean±SD)	Years A (Mean±SD)	Years B (Mean±SD)	C (Mean±SD)	D (Mean±SD)	E(Mean±SD)	Years Age (Mean±SD)
GES	49	6448.98 ± 1969.15	1.31 ± 1.37	1.18 ± 0.39	2.18 ± 1.52	4.02 ± 0.14	1.35 ± 0.48	12.16 ± 0.66
MES	49	6704.08 ± 2174.47	0.47 ± 0.53	1.18 ± 0.39	2.35 ± 1.38	4.02 ± 0.14	1.33 ± 0.47	12.31 ± 0.68			

GES - Gurukula Education system, MES - Modern education System, S - Salary of family, A - Education of father, B - Education of mother, (Education up to SSLC = 1, Graduation = 2, Postgraduation = 3, Professionials = 4) C - Occupation of father, D - Occupation of mother (Agriculture = 1, Business = 2, Academician = 3, Others = 4) E - Social setup (Rural = 1 Urban = 2) The results show no significant differences between GES and MES in all the demographic parameters (independent samples t test, *P* > 0.05). Differences between the GES and MES groups for levels of education of father, education of mother, occupation of father, occupation of mother, and social setup were assessed using X^2^ test and were found to be not significant (*P* > 0.05).

### Assessments

The SLCT consists of a test worksheet which specifies six target letters to be cancelled, and a ‘working section’ consisting of a 22 × 14 array of randomly arranged letters of the alphabets. Study participants were asked to cancel as many of the six target letters in the array as possible in the allotted time of 1:30 minutes. Subjects were told that there are two possible strategies: (i) canceling all six letters at once or (ii) selecting one target letter out of the six at a time. They were asked to choose whichever strategy suited them. They were also told that they could follow a horizontal, vertical, or a random path according to their choice. Scoring was carried out by a person blind to the details of the data. The total number of cancellations and wrong cancellations were scored and the net score calculated by subtracting wrong cancellations from total cancellations.[9] Each component measures a different quantity. The total number of cancellations is a measure of motor skill combined with cognitive function. The number of wrong cancellations is a measure of lack of focused attention and mental distractions. Net score is a measure of sustained attention.

### Masking

The demographic data concerning age, gender, and socio-economic status were collected by trained persons not involved in the design of the study. One-to-one matching of students was performed under the guidance and instruction of a trained statistician. All test assessments using the SLCT were conducted by trained persons under the supervision of a professional psychologist. Neither was involved in either the selection process or the study design. No teacher at either school was involved in making the assessments. There were no interactions between the GES and MES schools as they were in different locations more than 100 kilometres apart. Furthermore, no one at either school knew the identity of the other school.

### Intervention

The GES school used an educational program with integrated yoga practices, while the MES provided a conventional modern education program. The GES program included yoga postures (*asanas*), voluntary regulated breathing (*pranayama*), meditation (*dhyana*), recitation of mantras (*japa*), yogic prayers, worship (*puja*), and yogic games (a set of games which not only gives stimulation but also relaxation and calms the mind). The MES program included physical exercises, mathematical puzzles, music, prayer, and normal sports. The daily routine of the two schools match as shown in Table 2.

**Table 2:** Daily routine in the two residential schools

GES		MES	
Time*	Schedule	Time*	Schedule
5:00	Wake up	5:00	Wake up
5:30–5:45	Meditation and *pranayama*	5:00–5:50	Ablutions
5:45–6:15	Yogasanas	6:00–6:15	Prayer
6:15–6:45	Cleaning	6:15–6:45	Physical exercises
6:45–7:30	Ablutions	7:00–7:55	Self study
7:40–8:00	*Puja*		
8:30–9:15	Breakfast	8:00–9:15	Breakfast/cleaning
9:30–10:30	Vedic chanting	9:30–12.00	Sessions
10:30–12:45	Sessions	12.00–13:00	Music
13:00–14:30	Lunch	13:00–14:20	Lunch/rest
14:45–16:45	Sessions	14:30–16:30	Sessions
16:45–17:00	Snacks	16:45–17:00	Snacks
17:00–18:00	Tuning to nature	17:00–18:00	Tuning to nature
18:00–18:45	Yogic games	18:15–18:45	Games
18:45–19:00	Meditation and *pranayama*	18:45–19:00	Prayer
19:00–20:00	Self study	19.00–20.30	Self study
20:00–21.00	Dinner	20:30–21:15	Dinner
21:00–22.00	Self work	21:15–22:00	Self work
22.00	Lights off	22:00	Lights off

* 24-hour clock

### Data analysis

The predata of the two groups were compared using an independent samples ‘ *t* ’ test. The Kolmogorov test of normality showed that the predata were not normally distributed. Hence, nonparametric tests were used in the analysis. Within groups, the pre-post data were analyzed using the Wilcoxon signed ranks test, while between groups the pre-post data were analyzed using the Mann-Whitney U test. SPSS 10.0 was used for analysis.

## RESULTS

Both groups of students performed similarly on the pretest at the start of the academic year (predata). An independent sample’s ‘*t*’ test found no significant difference between the GES and MES groups. The Wilcoxon signed ranks test comparing the pre-post values within the groups showed that improvements in both groups were significant at *P* < 0.05. The Mann-Whitney U test used to compare results between the two groups showed a significant difference between the two groups (*P* < 0.05). The group average values ± SD for total scores, net scores, and scores for wrong cancellations of both GES and MES groups are given in Table 3.

**Table 3:** Scores on the six letter cancellation task

Variables	States	Groups				Between group significance*** on post scores
		GES		MES		
		Mean	SD	Mean	SD	
Total	Pre	41.82	3.187	40.67	2.45	0.001**
cancellations	Post	46.33*	3.68	43.08*	2.38	
Net scores	Pre	40.49	3.09	39.37	2.59	0.001**
	Post	45.39*	3.76	41.73*	2.49	
Wrong	Pre	1.39	0.64	1.41	0.73	0.001**
cancellations	Post	0.84*	0.72	1.35	0.75	

GES - Gurukula education system, MES - Modern education system

* *P* < 0.05, Wilcoxon signed ranks test, comparing pre and post values within groups.

** *P* < 0.05, Mann Whitney U test, comparing between roups.

*** Differences between groups on pre scores were not significant.

## DISCUSSION

Cancellation tasks require visual selectivity and a repetitive motor response.[10] They not only require sustained attention, but also visual scanning and activation and inhibition of rapid responses. The present study found a significant increase in sustained attention scores after the academic year for the GES group (*P* < 0.05), but the increase for the MES group did not reach significance.

The significant increase in net score for the GES group on the test suggests that the GES curriculum improves functioning of the right frontoparietal cortex mediating sustained attention.[2] Similarly, the significant increase in total score by the GES group suggests improvement in the frontal association areas, where the cognitive function guiding motor skills are located.[11] Decrease in wrong cancellations suggests that GES improves functions in the orbitofrontal area of the prefrontal cortex, which is hypothesized to mediate distraction avoidance.[12]

Several components in the GES curriculum could have contributed to the increase in the GES group’s sustained attention scores. Any kind of rhythmic resonance has the power to make the mind more relaxed and peaceful[13] and so improve attention span. Vedic *mantras* are highly rhythmic, and uniformly filled with resonance. Their daily chanting by the GES group may have been partly responsible for the observed increase in the group–s sustained attention scores.

Various papers have been published regarding growth of sustained attention through regular practice of meditation.[7] The GES group was engaged in daily practice of Gayatri *mantra* meditation throughout the year. This may also have contributed to the group’s observed increase in sustained attention.[14]

In addition to Gayatri meditation and vedic chanting, the GES group participated in yogic practices such as *asanas, pranayama*, and *puja*, which have the power to calm the mind, and bring the attention from past or future to the present moment. This may also have contributed to the observed growth of attention.

Reduced anxiety can improve performance on tasks requiring sustained attention[15] and yoga’s anxiety reducing effects[16] could also have facilitated this.

The students were assessed only twice during the entire academic year. No periodical assessments were conducted. One limitation of the study design, therefore, is that no immediate effect of GES was observed. Also, the single academic year time period of the study is not very long. Further studies assessing immediate effects of GES on sustained attention, and also assessing the whole time span of GES should be conducted. A further limitation of the study is that it does not evaluate how GES students utilize their improved attention span in their social and professional life after completing their education. Further studies could be designed to assess this.
